# Early Detection of Respiratory Depression Episodes with the Linshom Continuous Predictive Respiratory Sensor

**DOI:** 10.21203/rs.3.rs-8116603/v1

**Published:** 2025-12-01

**Authors:** Alberto Uribe, Ronen Feldman, Marco Echeverria-Villalobos, Talia Feldman, Ashish K. Khanna, Mahmoud Abdel-Rasoul, Richard D. Urman

**Affiliations:** The Ohio State University College of Medicine, Wexner Medical Center; Linshom Medical Inc; The Ohio State University College of Medicine, Wexner Medical Center; Linshom Medical Inc; Outcomes Research Consortium; The Ohio State University; Linshom Medical Inc

**Keywords:** Postoperative Respiratory Depression, Opioid-Induced Respiratory Depression, Continuous Respiratory Monitoring, Linshom Continuous Predictive Respiratory Sensor, Patient Safety in Post-Anesthesia Care, Early Detection Technology

## Abstract

**Purpose:**

The study aims to evaluate the effectiveness of the Linshom Continuous Predictive Respiratory Sensor (CPRS) for early detection of Respiratory Depression Events (RDE) in post-operative patients, with a focus on improving timely clinical intervention.

**Methods:**

In this prospective study, 472 adults undergoing non-cardiac surgery were monitored in the post-anesthesia care unit (PACU). Standard care included nurse monitoring, at a 1:1 nurse-to-patient ratio, supplemental oxygen, and continuous SpO_2_ monitoring. Simultaneously, the Linshom device continuously recorded respiratory parameters, capnography, and a second SpO2 signal (data blinded to staff). RDEs were defined by protocol and adjudicated by a 3-anesthesiologist panel provided with capnography data. Detection performance between Linshom Medical and SOC was evaluated using chi-square and McNemar’s tests.

**Results:**

The study monitored 472 patients; 132 (27.9%) experienced at least one respiratory depression event (RDE). The Linshom device detected 110 of these 132 events (83.3%), significantly outperforming SOC, which detected 59 events (44.7%). Linshom uniquely identified 73 events (55.3%) not detected by SOC, whereas 22 events (16.7%) were detected only by SOC, primarily through nurse interventions involving adjustments in oxygen administration. Linshom detected RDEs a mean of 11.06 (Standard Error ± 1.91) minutes earlier than SOC (p < 0.0001).

**Conclusion:**

The Linshom CPRS demonstrated superior sensitivity and earlier detection of postoperative respiratory depression compared to standard monitoring. Continuous respiratory monitoring with this technology may enhance patient safety and outcomes in the postoperative setting.

## Introduction

Postoperative respiratory complications, including OIRD, are common and undetected. (1) Patients often exhibit depressed respiration following surgery due to opioids used for pain management, sedation, ineffective airway clearance, or impaired gas exchange, such as during pneumonia, congestive heart failure (CHF), chronic obstructive pulmonary disease (COPD), and many other comorbidities. (2) Respiratory depression represents a serious patient safety concern that can lead to significant morbidity and mortality. It is traditionally defined by surrogate measures, such as hypoventilation with or without oxygen desaturation, and is often diagnosed by exclusion. A recent study, the PRediction of Opioid-induced respiratory Depression In patients monitored by capnoGraphY (PRODIGY) trial revealed that 46% of enrolled patients receiving opioids on the GCF experienced at least one RDE, which was defined as i) RR ≤ 5 breaths/min for ≥ 3minutes, or ii) SpO2 ≤ 85% for ≥ 3 minutes, or iii) end-tidal carbon dioxide (EtCO2) ≤ 15 or ≥ 60mm Hg for ≥ 3 minutes, or iv) apnea episode lasting > 30 seconds or any respiratory opioid-related adverse event. (1)

The literature indicates that the first 24 hours after surgery represent the highest risk period for RDEs, underscoring the need for careful monitoring during this period. (3) Respiratory monitoring in emergency rooms (ERs) and general care floors (GCFs) is crucial for the early detection of patient deterioration, particularly respiratory compromise. This can result in severe outcomes, such as respiratory failure or arrest, with a reported mortality of 40%. (4) Despite the clear benefits of continuous monitoring, the current standard of care for GCFs is to spot-check vital signs every 4 hours, leaving the patient unmonitored for approximately 96% of their time in the hospital wards. This increases the risk of undetected early warning signs of respiratory depression to nearly 90%. (4, 5) Sixty percent of patients experiencing rescue events (e.g., code blues) exhibit abnormal vital signs 4–6 hours prior to the event, and spot-check protocols often miss these signals. (6) Intermittent assessments are not only time-consuming but can be inaccurate and insufficient for capturing real-time changes in patient status. (7–9) The other component of SOC, pulse oximetry, detects oxygen desaturation; however, there is a clinically significant lag time between the onset of hypoventilation and measurable desaturation, which impedes early intervention. (10) Furthermore, the use of supplemental oxygen can mask hypoventilation by delaying desaturation, thereby rendering pulse oximetry less effective for early detection of OIRD. (11) A recent study conducted by Toften et al. reported that a substantial number of severe OIRDs occurred within 15 minutes of the last nursing check. This highlights the critical gap in patient safety posed by intermittent monitoring and underscores the need for continuous surveillance. (12)

Contrary to intermittent monitoring, continuous monitoring has been associated with double survival rates for in-hospital cardiac arrests, which are estimated to occur 290,000 times per year in the United States alone. (13) Furthermore, cardiorespiratory complications are the most common cause of 30-day postoperative mortality, and the Agency for Healthcare Research and Quality (AHRQ) rated postoperative respiratory failures as the fourth most common patient safety event in its 2015 report, using Patient Safety Indicator 11 (PSI 11) to identify and monitor this significant patient safety concern. (14, 15)

We hypothesized that the Linshom Continuous Predictive Respiratory Sensor (CPRS) detects postoperative RDEs earlier and more accurately than the current SOC (clinical observation + continuous pulse oximetry), in adult patients undergoing non-cardiac surgery. The study was designed to evaluate whether continuous, noninvasive bedside monitoring with the Linshom device provides a significant improvement over standard monitoring practices, which typically rely on intermittent or less comprehensive assessments. Capnography was used to validate the presence of RDEs; however, it was not included in the comparative analyses because it is not part of the PACU SOC.

## Methods

### Study Design and Population

After obtaining Institutional Review Board approval, we conducted this prospective Phase III study at the Ohio State University Wexner Medical Center. Research personnel enrolled 508 patients who underwent non-cardiac surgery. Before initiating patient enrollment, this study was registered on ClinicalTrials.gov (NCT05804175) on March 14, 2023, and adhered to the applicable guidelines of the Enhancing Quality and Transparency of Health Research (EQUATOR) initiative. It was conducted in accordance with the Principles of the Declaration of Helsinki. Research personnel screened and ensured that each subject enrolled met the inclusion criteria, and written informed consent was obtained from all participants. Patients were extensively screened in EPIC (IHIS) before being approached to minimize the number of screening failures and to follow the inclusion criteria of ≥ 18 years old undergoing non-cardiac surgery. All patients were admitted to the PACU immediately after surgery and stayed in the hospital overnight. They received supplemental oxygen via face mask in the PACU, were continuously monitored for SpO2, and received standard postoperative care.

### The Linshom Medical CPRS

Linshom Medical has developed the FDA-cleared CPRS, a small and inexpensive sensor and monitor that provides continuous, real-time tracking of critical indicators of ventilatory function. The CPRS is a thermodynamic device that includes a sensor with highly sensitive, rapid-responding thermistors that measure temperature change ten times per second during the inspiratory and expiratory cycles. Simultaneously, the device measures and controls for ambient temperature change via a proprietary control-loop algorithm. This cyclic temperature change generates a high-fidelity signal that enables continuous measurement of RR, rVt, rVe, SSLB, and I/E ratio. (16) This study aimed to determine if the Linshom CPRS can identify RDEs earlier and more frequently than the current SOC, which is comprised of clinical attention (1 nurse to 1 patient) and continuous pulse oximetry.

### Monitoring Protocol

Upon arrival at the PACU, each subject was fitted with an oxygen mask containing the Linshom sensor, which was connected to a Linshom monitor for data collection. A side-stream capnography line was attached to the same face mask, and the capnography data was collected on the Zoe Medical 740 SELECT^™^ monitor (Topsfield, MA). Additionally, two Masimo (Irvine, CA) pulse oximeters were applied to the same hand (non-NIBP arm), one connected to a hospital monitor (SOC) and the other to the Zoe Medical 740 SELECT^™^ monitor. The Linshom and 740 SELECT monitors collected time-synchronized data for all study parameters at one-second intervals. A clinical research assistant (CRA) attended the entire research stay in the PACU. Any clinical intervention (e.g., medications, oxygen delivery change, and stimulation upon detection of changes in the patient’s condition) performed by the PACU staff, and the time at which those interventions occurred, were recorded throughout the subject’s PACU stay by the CRA. Linshom Medical CPRS data collection was performed passively. At the same time, the patient was simultaneously monitored according to SOC. Clinical staff in the PACU were blinded to the Linshom CPRS data, as well as the additional pulse oximeter (non-SOC monitor) and capnography, to ensure that the collected study data would not influence clinical interventions. The non-SOC study parameters were not alarmed as part of the blinding protocol.

### Data Collection and Event Adjudication

After the subject’s data was collected in the PACU via the data acquisition system (DAQ), files were uploaded to a secure Dropbox server. Data from the Linshom Medical CPRS and the Zoe Medical 740 SELECT^™^ monitor were collected at one-second intervals and time-synchronized, and a graphical file was created for each patient. Graphs analyzed included overplots of Linshom CPRS RR and Capnography RR, Linshom CPRS RR and Capnography EtCO2, and Linshom CPRS RR and SpO2. Three anesthesiologists from the Ohio State University Wexner Medical Center independently reviewed graphical data. They examined every patient’s file in the study (N = 472), individually assessing the Linshom CRPS data correlation with Capnography data and comparing these data to pulse oximetry data and detailed CRA notes. Patients were categorized into those who experienced an RDE (N = 132) and those who did not, according to the study protocol definition. In this study, an RDE was defined according to modified PRODIGY criteria, with the capnography component removed because it is not part of the PACU standard of care (SOC). Accordingly, an RDE was identified when any of the following conditions occurred:

- Respiratory rate (RR) ≤ 5 breaths/min for ≥ 3 minutes, or
- Oxygen saturation (SpO_2_) ≤ 90% for ≥ 3 minutes, or
- Apnea episode lasting ≥ 30 seconds, or
- Any clinical intervention performed to minimize the risk of respiratory depression (e.g., stimulation, medication adjustment, or change in supplemental O_2_ delivery

These criteria reflect similar thresholds used in the PRODIGY trial, except for the exclusion of end-tidal CO_2_ (EtCO_2_) parameters. The complete RDE criteria are summarized in Table 1.

For each subject who experienced an RDE, the time (in minutes) of the first RDE detection was determined by a consensus reading of two of three expert physicians, who retrospectively reviewed the Linshom output in comparison to the SOC identification or intervention. The physicians, aided by graphical models of the data and detailed event notes taken by the CRAs, identified the times at which RDE was first detected, definitively determining which method or technology (Linshom, SOC, or both) was the fastest and most effective for identifying RDE.

### Statistical Analysis

Continuous demographic and clinical characteristics were summarized as mean (standard deviation) and compared between patients with and without a detected RDE using Student t-tests. Categorical demographic and clinical characteristics were summarized as frequency (percent) and compared between RDE groups using chi-square tests. The McNemar test was used to compare agreement in RDE detection between Linshom and SOC. A paired one-sided t-test and an alpha of 0.025 were used to test the null hypothesis that RDE detection time by Linshom (μL) will be longer than or equal to RDE detection time by SOC (μSOC):

$$H_{0}:\;\mu L\;-\;\mu \text{SOC}\;\geq \;0$$

$$H_{a}:\;\mu L\;-\;\mu \text{SOC}\;<\;0$$

For cases where only one method (Linshom or SOC) detected an RDE, that method was credited with detecting the RDE the shorter of 30 minutes before the method that did not detect it, or PACU discharge time. We further conducted sensitivity analyses assigning 25, 20, 15, and 10 minutes instead of 30 minutes. All statistical analyses were performed using SAS version 9.4 (SAS Institute, Cary, NC).

## Results

### Demographic data:

We enrolled 472 patients, with a median age of 61 years (IQR: 50–70) and a median BMI of 31.0 (IQR: 26.3–37.6). There were 333 female and 139 male patients (70.6% vs. 29.4%). Most patients had a risk stratification of ASA III (n = 298, 63.1%), followed by 160 with ASA II (33.9%), 10 with ASA IV (2.1%), and 4 with ASA I (0.8%). However, there was no significant association between the ASA stratification and the incidence of RDEs (p = 0.9810). Almost all patients received general anesthesia (99.8%) and only one received spinal anesthesia (0.2%). Other potential predisposing factors, such as BMI, active smoking, coronary artery disease, hypertension, and cardiovascular disease, were not associated with an increased incidence of RDEs (all p > 0.05). Patients experiencing RDEs were slightly older (median, 62 (IQR: 50.5, 97.0) vs 60 (IQR: 49.5, 69.0) years; p = 0.0933) and had a longer median hospital length of stay (1.3 vs 1.2 days; p = 0.0143) (Table 2).

### Incidence and Detection of RDEs

Of the 472 patients, 340 had no RDEs detected by any method (72.03%), 73 had RDEs detected only by Linshom CPRS (15.47%), 22 had RDEs detected only by the SOC (4.66%), and 37 had RDEs detected by both methods (7.84%). Among the 132 RDEs, 110 (83.33%) were detected by Linshom, compared with 59 (44.7%) by Standard of Care (SOC). (McNemar’s test p-value < 0.0001). (Table 3).

**Figure 1:**
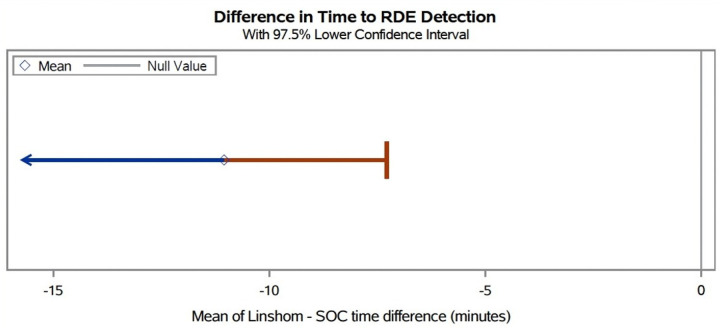
Difference in Time to RDE Detection The mean difference in paired detection times between Linshom and SOC (μL - μSOC) was −11.06 minutes (one-sided 97.5% upper confidence bound: −7.28; paired t-test p-value < 0.0001). For RDEs in which neither method detected the RDE, a value of either 30 minutes or the time to discharge from PACU (if <30 minutes) was used to represent the difference in detection time between methods.

The mean difference in paired detection times between Linshom and SOC (μL - μSOC) was − 11.06 minutes (one-sided 97.5% upper confidence bound: −7.28 paired t-test p-value < 0.0001). We further conducted sensitivity analyses assigning 25, 20, 15, and 10 minutes as the time difference, instead of 30 minutes, for instances in which only one method detected the RDE. Regardless of the time chosen, Linshom CPRS performed better than SOC (p < 0.0001), as shown in Fig. 1 and Table 4

**Figure 2:**
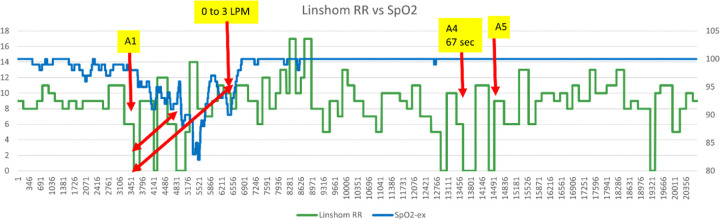
RDEs Occurring Without SpO_2_ Decline Following Supplemental Oxygen Increase A representative postoperative recording showing a Standard of Care (SOC) intervention triggered when SpO_2_ dropped below 90%, leading to an increase in supplemental oxygen from 0 to 3 L/min. Despite this intervention, four additional RDEs occurred—one lasting 67 seconds—without a significant change in SpO_2_ levels. This example illustrates how SpO_2_ monitoring alone may fail to detect ongoing respiratory depression in the presence of supplemental oxygen.

Notably, significant episodes of apnea were detected in patients receiving supplemental oxygen, without an accompanying decrease in SpO_2_, highlighting the limitations of relying solely on oxygen saturation for monitoring. As shown in Fig. 2, Linshom CPRS detected an RDE 2.4 minutes before SpO2 dropped below 90% and 4.85 minutes before a SOC change in supplemental oxygen. Despite the intervention, further apneas occurred (some prolonged at 48 and 67 seconds) while SpO2 remained near 100%.

**Figure 3:**
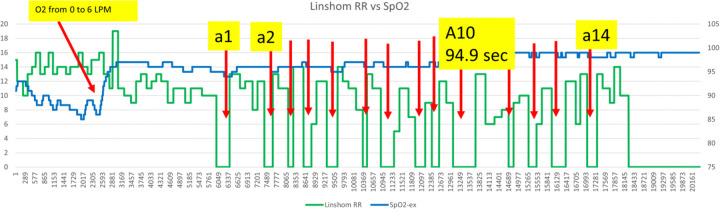
RDEs Occurring Without SpO_2_ Decline Following Supplemental Oxygen Increase – Example 2 RDEs Occurring Without SpO_2_ Decline After Increased Supplemental O_2_—Example 2. This figure depicts a postoperative patient monitored using the Linshom Continuous Predictive Respiratory Sensor (CPRS). Following a Standard of Care (SOC) intervention—an increase of supplemental oxygen from 0 to 6 L/min triggered by SpO_2_ decline—CPRS, highlighted by red arrows, detected numerous additional respiratory depression events (RDEs). Importantly, these RDEs, including one lasting 94.9 seconds, did not result in further significant declines in SpO_2_, demonstrating that SpO_2_ monitoring alone fails to detect ongoing respiratory depression when supplemental oxygen is administered.

In Fig. 3, a SOC intervention occurred when SpO2 fell below 90%, and supplemental oxygen was increased from 0 to 6 LPM. Fourteen additional RDEs occurred, with one lasting 94.9 seconds, and no significant change in SpO2 levels.

## Discussion

The findings of this study demonstrate that the Linshom CPRS significantly outperforms current SOC monitoring for detecting RDEs in postoperative patients. Notably, the Linshom device identified 83.3% of RDEs, compared to 44.7% detected by SOC, with a substantial proportion of events detected exclusively by the Linshom CPRS (55.3%) and only a small fraction detected exclusively by SOC (16.7%). This superiority in detection rate underscores the limitations of nurse observation, continuous pulse oximetry, and intermittent monitoring in general, and highlights the potential of continuous, noninvasive respiratory surveillance to enhance patient safety in high-risk clinical settings. Earlier notification of RDEs should allow HCPs a window for early action when the clinical intervention is relatively easy and inexpensive.

The true prevalence of respiratory depression has been historically underreported in clinical literature. Traditional estimates ranged from 0.3% to 21%, likely underestimating the actual incidence.(1) Other literature has reported OIRD incidence rates ranging from 0.15% to 1.1% among all post-surgical patients. These varying estimates reflect inconsistent taxonomy and methodology for identifying respiratory depression events, making it challenging to determine the true incidence. (3) The fact that 132 patients out of 472 (27.9%) experienced at least one RDE, a notably higher incidence than traditional literature estimates, aligns with the findings from the PRODIGY trial. (1) Linshom CPRS demonstrated much higher sensitivity for RDE detection than SOC, identifying most events earlier and more reliably. RDEs were detected 11.06 minutes earlier using the Linshom Medical CPRS compared to the standard of care. By identifying more RDEs than the SOC and doing so an average of 11 minutes earlier, the system facilitates timelier clinical intervention, when corrective actions are typically simpler and require fewer resources. The results of our study are consistent with a growing body of evidence that intermittent vital sign checks, which remain the standard on most hospital wards, are insufficient for the timely identification of respiratory depression episodes. The current SOC, typically consisting of periodic nursing assessments and continuous pulse oximetry, leaves patients unmonitored for extensive periods during their hospital stay and fails to capture early warning signs of respiratory depression. Capnography is the current “gold standard” in respiratory monitoring but has not yet reached the patient bedside due to its size, complexity, and cost. Spot-check visual assessments of RR have demonstrated lower sensitivity in detecting bradypnea or tachypnea. Pulse oximetry, another monitoring component of current SOC, measures oxygen saturation, with a lag time between the onset of signs of respiratory depression and the detection of oxygen desaturation. The early detection of subtle changes in the trend of sensitive and dynamic parameters of the ventilatory function, such as RR, SSLB, Vt, Ve, and I/E ratio, rather than the consequences of the RDE, makes the Linshom Medical CPRS a predictive mode of monitoring, rather than a reactive one, as are the currently available devices and SOC. (16, 18, 19)

Risk factors play a pivotal role in determining the incidence and severity of RDEs after surgery. Both patient-specific and perioperative factors contribute to the likelihood of these events, often acting synergistically to increase risk. Among patient-related risk factors, the most significant are comorbidities, including pre-existing cardiac disease, pulmonary disease, and obstructive sleep apnea (OSA), which are among the most critical predictors of RDEs. ASA physical status, specifically higher ASA class (≥ III), is independently associated with an increased risk of postoperative RDE, reflecting a greater overall disease burden and frailty. (23) Factors dependent on perioperative medication and surgery, such as the use of opioids, volatile anesthetics, and muscle relaxants, the type of surgery (laparoscopic vs open), the duration of surgery and anesthesia more than two hours, also potentiate the incidence of postoperative RDEs. (22, 24) Interestingly, in our study, neither patient-related risk factors (comorbidities, BMI, and genetic factors) nor intraoperative-related factors (type and duration of anesthesia, surgical technique, length of surgery) significantly increased the occurrence of RDEs.

We recognize several limitations of this study. First, the study was conducted at a single academic center, which may limit its generalizability to other settings, such as community hospitals or different patient populations. Second, only non-cardiac surgical patients were included; therefore, the results may not apply to other surgical populations, such as cardiac surgery, obstetrics, pediatrics, or ambulatory settings. Third, the study focuses on the detection of RDEs rather than on downstream clinical outcomes (e.g., ICU admission, rescue events, morbidity, and mortality). As of this writing, Ohio State University Wexner Medical Center is launching an NIH-funded clinical trial to assess these downstream clinical outcomes using the Linshom CPRS device. Fourth, although the sample size is robust overall, subgroup analyses (e.g., by comorbidity, anesthesia type) may be underpowered. Fifth, the monitoring period was limited to the PACU; however, the highest risk for RDE may extend beyond this period (e.g., on the general care floor).

## Conclusion

In this large, prospective study of postoperative patients, the Linshom CPRS demonstrated a significant improvement in the early, accurate detection of respiratory depression episodes (RDEs) compared with the current standard of care. The Linshom device detected, on average, 11.06 minutes earlier than standard monitoring, noting more RDEs, highlighting its potential to fill the critical gap left by nurse monitoring (despite a 1:1 nurse-to-patient ratio) through intermittent vital sign checks and continuous pulse oximetry, especially when supplemental oxygen use can mask hypoventilation. Notably, the increased sensitivity and quick response of Linshom CPRS did not reduce its specificity. Its portable, noninvasive design makes it ideal for routine postoperative monitoring. These results suggest that incorporating continuous respiratory monitoring devices, such as the Linshom CPRS, into standard postoperative care could significantly enhance patient safety by enabling earlier intervention and potentially reducing the risk of severe respiratory complications.

Future research should continue to investigate the impact of continuous respiratory monitoring on clinical outcomes, workflow integration, and cost-effectiveness across various patient groups and care settings. However, our findings offer strong evidence that the Linshom CPRS marks a significant step forward in detecting and managing postoperative respiratory depression, potentially establishing a new standard for perioperative respiratory monitoring.

## Figures and Tables

**Table 1:** Modified PRODIGY Definition of RDE Used in the Linshom Study.

RDE Definition
RR ≤ 5 bpm for ≥ 3 minutes, or
SpO2 ≤ 90% for ≥ 3 minutes, or
Apnea episode lasting ≥ 30 seconds, or
Any clinical intervention taken to minimize the risk of respiratory depression (e.g., medication, supplemental O2 change, or patient arousal)

**Table 2:** Subjects Demographic Characteristics (n = 472)

Factor	No. RDE (n = 340)	RDE (n = 132)	p-value
Age (years, median (IQR))	60.0 (49.5, 69.0)	62.0 (50.5, 73.0)	0.0933
Gender			0.6322
Female (%)	242 (71.2%)	91 (68.9%)	
Male (%)	98 (28.8%)	41 (31.1%)	
BMI (median (IQR))	31.3 (26.5, 37.7)	30.6 (25.7, 37.4)	0.1940
ASA Risk			0.9810
ASA I (%)	3 (0.9%)	1 (0.8%)	
ASA II (%)	117 (34.4%)	43 (32.6%)	
ASA III (%)	213 (62.6%)	85 (64.4%)	
ASA IV (%)	7 (2.1%)	3 (2.3%)	
Surgical Approach			0.5712
Laparoscopic (%)	174 (51.2%)	75 (56.8%)	
Open (%)	136 (40.0%)	46 (34.8%)	
Conversion (%)	2 (0.6%)	0 (0.0%)	
NA (%)	28 (8.2%)	11 (8.3%)	
Active Smoking (%)	28 (8.6%)	12 (9.3%)	0.8471
Comorbidities			
CVD (%)	143 (42.1%)	55 (41.7)	0.9382
CAD (%)	32 (9.5%)	6 (4.6%)	0.0852
HTA (%)	171 (50.4%)	76 (57.6%)	0.1638
Length of Surgery (h, median (IQR))	1.9 (1.2, 2.8)	2.2 (1.4, 3.1)	0.0948
Length of Anesthesia (h, median (IQR))	2.5 (1.8, 3.5)	2.8 (1.9, 3.8)	0.1033
PACU Stay (h, median (IQR))	2.0 (1.4, 2.8)	2.0 (1.5, 3.1)	0.2704
LOS (days, mean ± SD)	1.2 (0.4, 2.3)	1.3 (0.5, 2.4)	0.0143

**Table 3:** Comparison of RDE Detection by Linshom vs. SOC (n = 132 RDEs)

Detection Category	Number of RDEs	% of RDEs
Detected by Linshom (any)	110	83.3%
Detected by SOC (any)	59	44.7%
Detected by both Linshom and SOC	37	28.0%
Detected by Linshom but not SOC	73	55.3%
Detected by SOC but not Linshom	22	16.70%

**Table 4:** Time to Detection of RDEs: Linshom CPRS vs. Standard of Care

Assigned Time if Only One Method Detected	Mean Difference (Minutes)	Standard Error	One-sided 97.5% Upper Confidence Limit	p-value
30	−11.06	1.9078	−7.2811	< 0.0001
25	−9.4068	1.6605	−6.122	< 0.0001
20	−7.5048	1.3867	−4.7615	< 0.0001
15	−5.5842	1.0736	−3.4602	< 0.0001
10	−3.7674	0.7354	−2.3126	< 0.0001

## References

[1] Khanna AK, Bergese SD, Jungquist CR, Morimatsu H, Uezono S, Lee S, Prediction of Opioid-Induced Respiratory Depression on Inpatient Wards Using Continuous Capnography and Oximetry: An International Prospective, Observational Trial. Anesth Analg. 2020;131(4):1012–24. 10.1213/ANE.0000000000004788. 32925318 PMC7467153

[2] Morris TA, Gay PC, MacIntyre NR, Hess DR, Hanneman SK, Lamberti JP, Respiratory compromise as a new paradigm for the care of vulnerable hospitalized patients. Respir Care. 2017;62(4):497–512. 10.4187/respcare.05021. 28341777

[3] Gupta RK, Edwards DA. Monitoring for opioid-induced respiratory depression. APSF Newsl. 2018;32(3):70–2.

[4] Andersen LW, Berg KM, Chase M, Cocchi MN, Massaro J, Donnino MW, Acute respiratory compromise on inpatient wards in the United States: Incidence, outcomes, and factors associated with in-hospital mortality. Resuscitation. 2016;105:123–9. 10.1016/j.resuscitation.2016.05.014. 27255952

[5] Spratt GK. Patient Monitoring: Assessing Patient Risk of Opioid-Induced Respiratory Compromise. RT: J Respiratory Care Practitioners. 2019;32(2).

[6] Andersen LW, Kim WY, Chase M, Berg KM, Mortensen SJ, Moskowitz A, The prevalence and significance of abnormal vital signs prior to in-hospital cardiac arrest. Resuscitation. 2016;98:112–7. 10.1016/j.resuscitation.2015.08.016. 26362486 PMC4715919

[7] Paul JE, Buckley N, McLean RF, Antoni K, Musson D, Kampf M, Hamilton acute pain service safety study: using root cause analysis to reduce the incidence of adverse events. Anesthesiology. 2014;120(1):97–109. 10.1097/ALN.0b013e3182a76f59. 24398730

[8] Khanna AK, Bergese SD, Jungquist CR, Morimatsu H, Uezono S, Lee S, . Prediction of opioid-induced respiratory depression on inpatient wards using continuous capnography and oximetry: an international prospective, observational trial. Anesth Analgesia. 2020;131(4):1012–24. 10.1213/ANE.0000000000004788. PMC7467153 32925318

[9] Sun Z, Sessler DI, Dalton JE, Devereaux P, Shahinyan A, Naylor AJ, Postoperative hypoxemia is common and persistent: a prospective blinded observational study. Anesth Analgesia. 2015;121(3):709–15. 10.1213/ANE.0000000000000836. PMC4825673 26287299

[10] Ayad S, Khanna AK, Iqbal SU, Singla N. Characterisation and monitoring of postoperative respiratory depression: current approaches and future considerations. Br J Anaesth. 2019;123(3):378–91. 10.1213/ANE.0000000000004788. 31331649

[11] Keidan I, Gravenstein D, Berkenstadt H, Ziv A, Shavit I, Sidi A. Supplemental oxygen compromises the use of pulse oximetry for detection of apnea and hypoventilation during sedation in simulated pediatric patients. Pediatrics. 2008;122(2):293–8. 10.1542/peds.2007-2385. 18676546

[12] Toften S, Kjellstadli JT, Kværness J, Pedersen L, Laugsand LE, Thu OK. Contactless and continuous monitoring of respiratory rate in a hospital ward: a clinical validation study. Front Physiol. 2024;15:1502413. 10.3389/fphys.2024.1502413. eCollection 2024. 39665054 PMC11631942

[13] Andersen LW, Holmberg MJ, Berg KM, Donnino MW, Granfeldt A. In-hospital cardiac arrest: a review. JAMA. 2019;321(12):1200–10. 10.1001/jama.2019.1696. 30912843 PMC6482460

[14] Stocking JC, Utter GH, Drake C, Aldrich JM, Ong MK, Amin A, Postoperative respiratory failure: an update on the validity of the Agency for Healthcare Research and Quality Patient Safety Indicator 11 in an era of clinical documentation improvement programs. Am J Surg. 2020;220(1):222–8. 10.1016/j.amjsurg.2019.11.019. 31757440 PMC10091853

[15] Garcia N. The Use of the Agency Healthcare Research and Quality Patient Safety Indicator 11 Toolkit to Decrease Postoperative Respiratory Failure. 2023.

[16] Wadhwa V, Gonzalez AJ, Selema K, Feldman R, Lopez R, Vargo JJ. Novel device for monitoring respiratory rate during endoscopy-A thermodynamic sensor. World J Gastrointest Pharmacol Ther. 2019;10(3):57–66. 10.4292/wjgpt.v10.i3.57. 31598389 PMC6783685

[17] Overdyk FJ, Carter R, Maddox RR, Callura J, Herrin AE, Henriquez C. Continuous oximetry/capnometry monitoring reveals frequent desaturation and bradypnea during patient-controlled analgesia. Anesth Analg. 2007;105(2):412–8. 10.1213/01.ane.0000269489.26048.63. 17646499

[18] Brady WJ, Gurka KK, Mehring B, Peberdy MA, O’Connor RE. In-hospital cardiac arrest: impact of monitoring and witnessed event on patient survival and neurologic status at hospital discharge. Resuscitation. 2011;82(7):845–52. 10.1016/j.resuscitation.2011.02.028. 21454008

[19] Kor JJ, Sprung J, Khanna AK, Weingarten TN. Continuous monitoring detected respiratory depressive episodes in proximity to adverse respiratory events during the PRODIGY trial. J Patient Saf. 2022;18(8):738–41. 10.1097/PTS.0000000000001003. 35405725 PMC9698081

[20] Sun Z, Sessler DI, Dalton JE, Devereaux PJ, Shahinyan A, Naylor AJ, Postoperative Hypoxemia Is Common and Persistent: A Prospective Blinded Observational Study. Anesth Analg. 2015;121(3):709–15. 10.1213/ANE.0000000000000836. 26287299 PMC4825673

[21] Taenzer AH, Pyke JB, McGrath SP, Blike GT. Impact of pulse oximetry surveillance on rescue events and intensive care unit transfers: a before-and-after concurrence study. Anesthesiology. 2010;112(2):282–7. 10.1097/ALN.0b013e3181ca7a9b. 20098128

[22] Pennett A. Risk factors for respiratory depression in postoperative orthopedic trauma patients. University of Pittsburgh; 2014.

[23] Laporta ML, Sprung J, Weingarten TN. Respiratory depression in the post-anesthesia care unit: Mayo Clinic experience. Bosnian J basic Med Sci. 2021;21(2):221. 10.17305/bjbms.2020.4816. PMC7982067 32415817

[24] Sigona A, Richman DC. Identifying and reducing risks of postoperative pulmonary complications. J Oral Maxillofacial Anesth. 2023;2. 10.21037/joma-23-20.

